# Feasibility and Efficiency of a Novel Bolus Calculator (IF-DIABETE) for Patients With Type 1 Diabetes: A Nonrandomized Single-Arm Pilot Study

**DOI:** 10.7759/cureus.12646

**Published:** 2021-01-12

**Authors:** Siham Rouf, Najwa Rbiai, Kaoutar Baibai, Jamal Berrich, Toumi Bouchentouf, Mohammed Rahmoun, Naima Abda, Hanane Latrech

**Affiliations:** 1 Diabetology and Endocrinology, Mohammed VI University Hospital, Medical School, Mohamed the First University, Oujda, MAR; 2 Research Laboratory in Applied Sciences, National School of Applied Sciences, Mohamed the First University, Oujda, MAR; 3 Laboratory of Epidemiology, Clinical Research and Public Health, Mohammed the First University, Oujda, MAR

**Keywords:** telemedicine, type 1 diabetes, if-diabete, metabolic control, hypoglycemia

## Abstract

Background

The IF-DIABETE system is an insulin bolus dose support, considered as the first bolus calculator dedicated to people with type 1 diabetes, designed in the Arabic language, and adapted to the large Arabic food culture. Our aims were to assess the proof of concept and efficiency of the IF-DIABETE system in improving clinical and metabolic outcomes in individuals with type 1 diabetes.

Methods

This is a prospective nonrandomized single-arm pilot study. Our patients used the IF-DIABETE smartphone application as a novel bolus calculator. Over six months period of the study, the primary outcome considered was hemoglobin glycated (HbA1c), and we identified hypoglycemic events, body mass index (BMI), and the frequency of blood glucose measurements as secondary outcomes.

Results

Twenty-one patients with type 1 diabetes were enrolled. The average age was 21 ± 3 years. Over a six months’ period of the study, the mean HbA1c level decreased from 8.3 ± 0.8% to 7.0 ± 0.5 % with a reduction in mild hypoglycemic events’ frequency from 5 ± 3 to 1 ± 0.7 episodes/3 months. We did not observe any change in BMI and the frequency of the blood glucose testing improved from 2 ± 0.5 to 5 ± 1 tests per day.

Conclusion

The IF-DIABETE system was safe and effective to support individuals who have type 1 diabetes to improve their metabolic control. At six months, patients were able to improve their glycemic control without increasing the risk of hypoglycemic events.

## Introduction

Tight glycemic control is the main purpose of the management of type 1 diabetes. The basal-bolus regimens with multiple daily injections or insulin pump in addition to the educational programs such as flexible insulin therapy (FIT) were recommended as the optimum therapy for the patient with type 1 diabetes to achieve better glycemic control and prevent microvascular complications (nephropathy, neuropathy, and retinopathy) [1, 2]. Nevertheless, reaching the recommended glycemic control remains challenging across all age groups and exposed to a higher risk of hypoglycemia [3].

The FIT was adopted as a five-day inpatient training by a team from Dusseldorf to teach individuals with type 1 diabetes how to adapt their fast-acting insulin doses to their carbohydrate intakes and physical activity [4]. However, despite all these therapeutic approaches, metabolic control is not always obtained [5].

The supposed reasons for this unsatisfactory glycemic control can be illustrated by the difficulties to “do with” the constraints of the chronic diabetic disease and to respect correctly the daily rules necessary to determine the doses of the fast-acting insulin, which leads to injections of inappropriate doses consequently responsible for poor glycemic control. All these described constraints create challenges for patients with type 1 diabetes to achieve good glycemic control [5, 6].

Telemedicine in diabetes has emerged as a promising approach for improving outcomes in people with type 1 diabetes. Benefits of this option have been demonstrated in several studies [7-10] and many sophisticated systems have been developed providing patients the opportunity to assist them in their daily determination of the doses of the prandial insulin according to pre-meal glucose, carbohydrate intake, and physical activity [11, 12].

Considering the large Arabic food culture, people with type 1 diabetes in many Arabic countries usually find difficulties in using common diabetes smartphones application. The IF-DIABETE system was developed to support these patients in their daily calculation of the fast-acting insulin according to pre-meal glucose level, carbohydrates intake, and physical activity with the possibility to record their blood glucose tests in electronic diaries.

We aimed to assess the proof of concept and the effectiveness of the IF-DIABETE system in improving clinical and metabolic outcomes in patients with type 1 diabetes before starting a randomized controlled study over 18 months comparing the IF-DIABETE system with a control group.

## Materials and methods

Study design

We performed a six-month, prospective, nonrandomized, single-center, open-label pilot study at the Mohammed VI University Medical Center in Oujda, Morocco, between June 2019 and January 2020. All patients gave their informed consent to be included in the study. The data was collected anonymously and the analysis was performed with a strictest confidentiality. Ethical review and clearance were gathered from the Medical Ethics Committee at the Faculty of Medicine, Mohamed the First University, Oujda.

Study population

Twenty-one type 1 diabetes patients were enrolled. Inclusion criteria were patients aged 18 to 60 years, diagnosed with type 1 diabetes (as defined by the American Diabetes Association) for at least six months and who were treated either by multiple daily injections (short-acting insulin and long-acting insulin) or by pump therapy [13]. All patients were familiar with flexible intensive insulin therapy. They were inadequately controlled (HbA1c between 7.5% and 10%).

The exclusion criteria were patients with type 2 diabetes, pregnancy, difficulty to ensure a minimum of three self-measured blood glucose tests per day, psychiatric disorders, unmotivated patients, and the presence of severe macrovascular or microvascular complications. We also excluded patients who had technical difficulties.

Study protocol

After inclusion, the therapeutic education team provided participants two hours of training in our department to explain the functionality of the application. Patients were called after one week to be sure there were no technical problems. All participants received the same program with individual consultations at three and six months. At each consultation, basal insulin and carbohydrate ratio were adjusted as necessary.

Outcomes

All data were collected initially then at three months and six months during the study period. The effectiveness of the application was regularly judged by the quarterly HbA1C %, the frequency of self-monitoring blood glucose and the frequency of hypoglycemic events.

The primary efficacy outcome was the change in the HbA1c level from baseline to end point. It was measured by high-performance liquid chromatography (HPLC) on the hospital site, which permits measurement of HbA1c values between 2.5% and 17%.

Secondary outcomes were the frequency of hypoglycemic events, assessed in number of hypoglycemic episodes per month, BMI and the frequency of blood glucose measurements.

The mild hypoglycemia was defined as self-treated symptomatic hypoglycemia or a blood glucose level of <70 mg/dl and severe hypoglycemic events were defined as any episode requiring help from a third party [14].

IF-DIABETE system architecture

**Figure 1:**
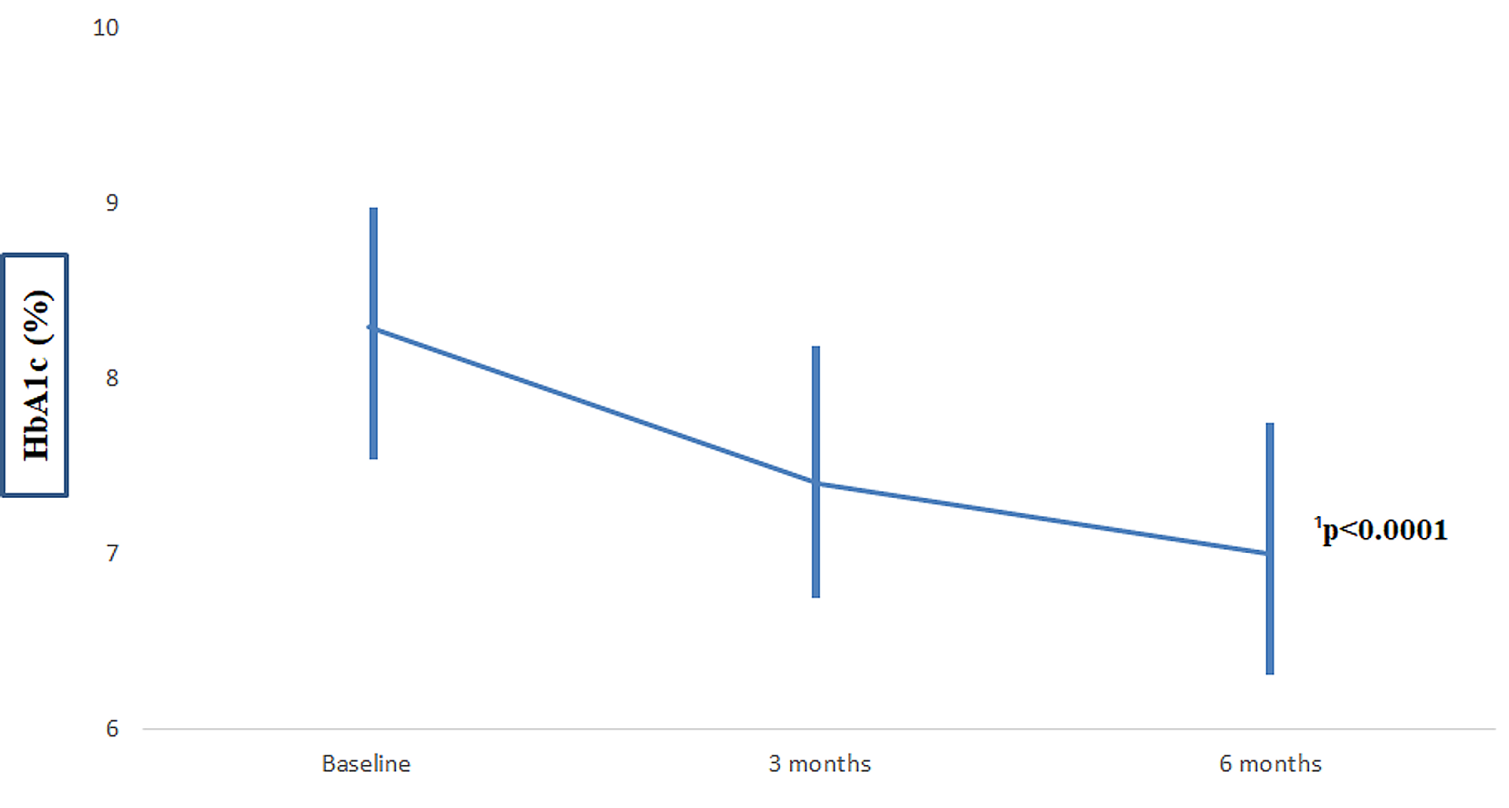
Evolution of the average of HbA1c between the installation of IF-DIABETE system and the end of the study. ¹Mixed linear model corrected by the Bonferroni test

The IF-DIABETE platform couples a diabetes smartphone application and a website. The smartphone application is available on Google play store. The daily access to the application does not require access to internet connection and is 100% free of charge (Figure 1).

The website contains a database of patients, each one has a patient index (IP) and a password that is obtained after the creation of a file and the introduction of data relating to each patient. This password allows the patient to log in to the application on his smartphone.

The digital file includes the socio-economic data of patients, the medical file with the history of the disease, the diabetes chronic complications, the parameters of the flexible insulin therapy (insulin to carbohydrate ratio and insulin sensitivity factor), and the glycemic targets.

The website also includes a detailed electronic nutritional booklet that we have created, which includes different foods and their carbohydrate content, taking into account the specificity of Arabic food traditions.

The patient can log in to the application to calculate the amount of carbohydrate in his meal and determine the dose of the fast-acting insulin adjusted to the pre-prandial glucose level, the carbohydrate intake of meals and physical activity.

This calculation is based on the concepts of FIT including the insulin to carbohydrate ratio and the “insulin sensitivity factor”.

The insulin to carbohydrate ratio was determined from insulin requirements of fast-acting insulin used in the conventional regimen (the insulin units needed for 10g of carbohydrates), and the “insulin sensitivity factor” which is a dose of short-acting insulin individualized according to the insulin sensitivity of each patient (the effect of 1 unit of fast-acting insulin on blood glucose lowering) determined by using the common method of “1800 Rule” which is 1800/Total Daily Dose (TDD) of long- and short-acting insulin = glucose rate in mg/dL decreased by administering 1 unit of short-acting insulin.

Besides, the application allows other functions such as the reminder of blood glucose test 4 hours after the meal. This reminder is triggered automatically after confirmation of the injection of the dose of insulin. The daily recording of blood glucose levels makes it possible to generate PDF reports, replacing the written blood glucose diaries.

Finally, the patient has the opportunity to personalize the food list by adding references to the original list according to his own eating habits.

This telemedicine system (IF-DIABETE) was developed and created in partnership with the Research Laboratory in Applied Sciences of the National School of Applied Sciences, Oujda. It has brought together doctors and computer scientists for daily work with regular meetings over a period of two years.

Statistical analysis

All statistical calculations were performed with the Statistical Package for Social Sciences (SPSS), V21.0 (IBM Corp., Armonk, NY). The different data were normally distributed and presented as means ± SD. The comparison of the outcomes including the glycated hemoglobin, the rate of mild hypoglycemia, BMI and the frequency of blood glucose measurements was performed between the different follow-up time points by using the mixed linear model corrected by the Bonferroni test. A p-value <0.05 was considered statistically significant.

## Results

Study participants

Twenty-one patients with type 1 diabetes were enrolled and followed up. There were 17 girls and four boys, sex ratio F/M: 4.2. The average age at inclusion was 21 ± 3 years. The average duration of diabetes was 5 ± 4 years. The initial mean HbA1c value was 8.3 ± 0.8%.

Effect on HbA1c

At the end of the follow-up period of six months, we observed a significant reduction of the HbA1c value by 1.3% moving from 8.3 ± 0.8% to 7.0 ± 0.5% (p =< 0.0001). However, participants with an HbA1c over 8% decreased greatly their baseline HbA1c value from 9.1 ± 0.7% to 7.1 ± 0.5% (p = 0.001).

Effect on the frequency of hypoglycemia

Prior to the study, 71.4% of patients had the experience of mild hypoglycemia. Nevertheless, no patient had suffered from severe hypoglycemia.

At the end of the study, the frequency of mild hypoglycemia decreased significantly five-fold moving from 5 ± 3 to 1 ± 0.7 episodes/3 months (p =< 0.0001) (Table 1).

**Table 1:** Outcomes in the entire group at 3 and 6 months (n = 21) Note: Data are presented as the number of the mean (SD).

	Baseline	3 months	6 months	p
Hemoglobin glycated (%)	8.3 (0.8)	7.4 (0.7)	7.0 (0.5)	<0.0001
Body mass index (kg/m²)	21 (3)	21 (2)	21 (2)	NS
Blood glucose tests (per day)	2 (0.5)	4 (1)	5 (1)	<0.0001
Frequency of mild hypoglycemia (episodes/3 months)	5 (3)	2 (1)	1 (0.7)	<0.0001

We were able to conclude that better metabolic control was not associated with an increased risk of hypoglycemic events.

Effect on BMI and the frequency of blood glucose tests

The initial average BMI was 21 ± 3 kg/m², which is remained unchanged. However, we observed a significant improvement in the frequency of the capillary blood glucose tests moving from 2 ± 0.5 to 5 ± 1 measurements per day (p =<0.0001).

## Discussion

Various telemedical programs have improved metabolic control in adult patients with type 1 diabetes [15-17]. The results in this report demonstrated the proof of concept and efficiency of the IF-DIABETE system to improve glycemic control in patients with type 1 diabetes besides reducing the risk of hypoglycemic events. The basic HbA1c value decreased significantly by 1.3% going from 8.3% to 7.0% at the end of the study. This result was clinically significant since the Diabetes Control and Complications Trial reported that a reduction of 1% in HbA1c reduced the risk of microvascular disease by 21%-48% [18].

Tight metabolic control was associated with a higher risk of hypoglycemia events in type 1 diabetic patients [3, 19]. In this report, the metabolic improvement was associated with a lower rate of hypoglycemic events and during the follow-up, the frequency of mild hypoglycemia declined from 5 to 1 episodes/3 months at six months. This present finding is valuable, considering the negative impact of hypoglycemia on quality of life, clinical outcomes [20,21], and cost of diabetes care [22].

All patients improved significantly the rate of their daily blood glucose testing (p =< 0.0001). Several telecare studies reported that metabolic improvement was traditionally correlated with an increase in the frequency of blood glucose measurements, which was comparable to our results [23, 24].

Flexible insulin therapy is one of the fundamental elements in the management of type 1 diabetes. This therapeutic approach is proven to be effective in reducing HbA1c in type 1 diabetic patients in many previous studies [5, 25]. We remind that all our patients had previously received flexible insulin therapy training in our department. The knowledge acquired during this training was applied in everyday life.

However, effective management of type 1 diabetes needs closer assistance from caregivers, which can be facilitated by contemporary telemedical systems [11,12]. The diabetes smartphone applications are not supposed to replace patient-care professional contact but rather to support type 1 diabetic patients in their diabetes self-management.

The IF-DIABETE system was developed to support poorly controlled type 1 diabetic patients in many Arabic countries previously educated for flexible insulin therapy. This system allows patients to manage their daily glycemic monitoring and support the therapeutic approach of flexible insulin therapy to obtain better metabolic control.

Many previous diabetes telemedicine studies reported educational sessions organized for the patients at the start, on how to use the investigated technology during 15 minutes session [26], one or six hours [23, 27, 28], and even one day [29, 30]. In our unit, the therapeutic education team provided participants two hours of training to explain the functionality of the application. These education sessions were organized in order to facilitate the use of the IF-DIABETE application. During the follow-up, patients reported that the design and the functionality of this application were intuitive and user-friendly.

To our knowledge, this is the first diabetes smartphone application designed in the Arabic language in order to assist patients with type 1 diabetes to improve their metabolic control. This platform was adapted to the Arabic food culture. All participants were satisfied with the IF-DIABETE application, which allowed them to define easily the doses of the fast-acting insulin in a few minutes, according to the carbohydrates in the meal, pre-meal glucose value, and physical activity. The possibility to record and not writing at all the blood glucose value was the additional valuable benefit for our diabetic patients.

In order to upgrade our Telemedicine system, the IF-DIABETE system will be equipped with an additional function that generates alerts notifying care professionals if blood glucose level is over the target range. This function should enhance the effectiveness of the system and allows physicians to intervene as soon as possible. In general, patients require more contact and support from care professionals to improve their metabolic control and they become empowered to manage perfectly their diabetes.

Despite that the present study was a short, nonrandomized pilot study without a group control. The results are encouraging showing that the IF-DIABETE system is useful for type 1 diabetic patients with particular food culture. A further report in the form of a randomized controlled trial over 18 months is underway to evaluate whether the IF-DIABETE system is superior to mental calculations considering the complexity of Arabic dishes.

## Conclusions

The application of telemedicine applied to patients with type 1 diabetes is an effective strategy, providing positive results on the metabolic improvement, treatment adherence, and the acquisitions of knowledge for patients with type 1 diabetes; and why not this approach could be adopted in Arabic countries with adaptive telemedical support to help patients with type 1 diabetes to improve their glycemic balance in order to avoid developing chronic complications that can alter their lives even at an early age.
